# Pelvic venous disorders: Objective hemodynamic signature from quantitative two-dimensional phase-contrast magnetic resonance imaging

**DOI:** 10.1016/j.jvsv.2026.102616

**Published:** 2026-09-05

**Authors:** Shih-Chung Wang, Chen-Yu Li, Chih-Chen Kao, Yin-Chen Hsu, Chung-Yuan Lee, Teng-Yao Yang, Sheng-Ya Chen, Yen-Hsi Lai, Yu-Hui Lin, Min Yi Wong, Chien-Wei Chen, Yao-Kuang Huang

**Affiliations:** a Department of Diagnostic Radiology, ChiaYi Chang Gung Memorial Hospital, Chiayi, Taiwan; b College of Medicine, Chang Gung University, Taoyuan, Taiwan; c Department of Medical and Radiology, Shu-Zen Junior College of Medicine and Management, Kaohsiung, Taiwan; d Department of Finance, National Taichung University of Science and Technology, Taichung, Taiwan; e Division of Thoracic and Cardiovascular Surgery, ChiaYi Chang Gung Memorial Hospital, Chiayi, Taiwan; f Department of Obstetrics and Gynecology, ChiaYi Chang Gung Memorial Hospital, Chiayi, Taiwan; g Department of Cardiology, ChiaYi Chang Gung Memorial Hospital, Chiayi, Taiwan; h Division of Thoracic and Cardiovascular Surgery, Chiayi Hospital, MOHW, Chiayi, Taiwan

**Keywords:** MRI, Noncontrast, Phase contrast, Pelvic congestion, Venous disease, PeVD

## Abstract

**Objective:**

The lack of objective hemodynamic criteria for diagnosing pelvic venous disorders (PeVD) limits interdisciplinary consensus among vascular surgery, radiology, phlebology, and gynecology societies. We applied noncontrast two-dimensional phase-contrast magnetic resonance imaging (2D PC-MRI) to quantify pelvic-lower-limb venous flow and assess diagnostic performance.

**Methods:**

A retrospective cross-sectional study was conducted in 428 women who underwent 2D PC-MRI between 2017 and 2024. Participants included PeVD (n = 138), healthy controls (n = 39), and symptomatic women without PeVD (n = 251). An additional subset of symptomatic women with PeVD who underwent venous intervention (n = 26) was analyzed. Hemodynamic parameters included stroke volume (SV), mean flux (MF), stroke distance (SD), mean velocity (MV), and ankle interstitial pixel-shift signals.

**Results:**

Patients with PeVD demonstrated a consistent hemodynamic signature marked by increased central flow, reduced superficial flow, and left gonadal vein (LGV) reflux. Compared with healthy controls, patients with PeVD had higher right external iliac vein, SV, and MF, lower left great saphenous vein (GSV), SV, and MF, and pronounced LGV abnormalities (SV, 1.29 vs 0.54 mL; MF, 1.50 vs 0.60 cm^3^/s; SD, −0.50 vs 2.14; MV, −0.49 vs 2.39; all *P* = .0001). Popliteal vein flow was altered, and ankle signals were elevated. Receiver operating characteristic analysis identified left GSV hypoflow (area under the curve, 0.69-0.71) and LGV SD/MV (area under the curve, ∼0.70-0.71) as the most informative markers.

**Conclusions:**

Noncontrast 2D PC-MRI yields objective assessment of morphology and segment-specific hemodynamics in lower-body venous system. LGV reflux and GSV hypoflow are the most informative markers of PeVD.

**Article Highlights**

• **Type of Research:** A single institution, retrospective cross-sectional study of 428 women evaluating the diagnostic performance of noncontrast two-dimensional phase-contrast magnetic resonance imaging in pelvic venous disorders (PeVD)
• **Key Finding:** Patients with PeVD exhibit a consistent hemodynamic signature characterized by significant left gonadal vein reflux and reduced superficial flow in the great saphenous veins. Left gonadal vein stroke distance and mean velocity are high-performing markers (area under the curve, ∼0.71) for differentiating PeVD from healthy individuals.
• **Take Home Message:** Noncontrast two-dimensional phase-contrast magnetic resonance imaging provides objective, segment-specific hemodynamic metrics that facilitate physiology-based diagnosis and lesion-directed treatment planning without the need for radiation or contrast media.

Over 15% of women aged between 20 and 50 years' experience a pelvic venous disorders (PeVD).^1^ Furthermore, 30% to 45% of women with chronic pelvic pain (CPP) may have symptoms related to venous disorders.^2^^,^^3^ Pelvic venous science has advanced rapidly in recent years, driven by improvements in diagnostic techniques and an expanding understanding across phlebology, interventional radiology, and vascular surgery societies.^4^, ^5^, ^6^ However, gynecology and other specialties have paid insufficient attention to it.^7^^,^^8^ A key reason is the lack of an objective, effective diagnostic tool for venous disease that enables clear communication between patients and specialists.

Ultrasound study (US), including intravenous, transabdominal, and transvaginal approaches, is the most widely used noninvasive modality for detecting venous disorders, and a global consensus on its role in diagnosing PeVD has gradually emerged.^9^^,^^10^ However, the clinical utility of US is often limited by body habitus and bowel gas, which can prevent the visualization of the iliac veins, retroperitoneal structures, paraspinal regions, and the inferior vena cava (IVC). Moreover, US visualization is subjective, and its hemodynamic assessment can vary between operators. Additional evaluation with cross-sectional imaging modalities, such as contrast-enhanced magnetic resonance angiography (CE-MRA) and computed tomography venography, is often selected to rule out pelvic pathologies. Although CE-MRA and computed tomography venography offer more objective diagnoses and are more suitable for examining pelvic vessels and the IVC, they provide mostly morphological insights required contrast media injection.^3^ Recent vascular surgery literature has emphasized that PeVD represents a heterogeneous spectrum of venous reflux and obstruction involving the ovarian, internal iliac, and pelvic venous plexus systems rather than an isolated ovarian vein disorder.^11^, ^12^, ^13^ Contemporary studies have demonstrated that symptom severity is associated not only with venous reflux but also with the extent of pelvic venous congestion and network involvement.^11^^,^^13^^,^^14^ Recent reviews have further highlighted the lack of standardized severity grading systems and objective hemodynamic outcome measures in PeVD, underscoring the need for imaging modalities capable of evaluating both pelvic venous anatomy and flow characteristics.^12^^,^^14^^,^^15^ Therefore, advanced imaging techniques that provide both anatomical and hemodynamic assessment may improve patient selection, treatment planning, and objective evaluation of treatment response in PeVD.

Noncontrast MRA (NC-MRA), which incorporates various contrast mechanisms, can be used to evaluate PeVD. Our institution has used NC-MRA with a gated three-dimensional (3D) turbo spin-echo short tau inversion recovery (TSE-STIR) sequence since 2017 for the diagnosis of lower-extremity venous disorders.^16^ In a previous study, this technique was determined to be effective when used in combination with US.^17^ The 3D TSE-STIR sequence enables the visualization of venous structures [magnetic resonance venography (MRV)] during the systolic phase of the cardiac cycle because arterial blood flows rapidly and creates flow voids, while STIR suppresses additional background signals from fat and bone. The entire venous anatomy of the lower extremities, particularly the low-flow superficial venous system and pelvic collaterals, can be clearly observed through 3D imaging without the use of a contrast medium or radiation. Another technique, also known as quantitative phase-contrast magnetic resonance imaging (PC-MRI; QFlow; Medis Medical Imaging Systems B.V.), enables the quantification of PC parameters in regions of interest (ROI) on a 2D plane (2D PC-MRI).^18^^,^^19^ At our institution, an MRI protocol combining gated 3D TSE-STIR MRV with quantitative 2D PC-MRI has become the standard preoperative assessment for lower-body venous disease and has proven effective, even in the pelvic region^20^ (Fig 1). Using this objective venous imaging modality, we collected anatomical information of the pelvic veins (including diameter, venous morphology, and the presence of collateral circulation) and hemodynamic data (such as flow volume, velocity, and the presence of reflux). Combined with clinical symptoms (eg, CPP and dyspareunia), these parameters were used to identify patients with PeVD. Conversely, patients without significant pelvic venous abnormalities were classified as either isolated lower-extremity venous disease or nonvenous patients.^4^^,^^21^ In this study, we analyzed quantitative 2D PC-MRI data from patients with PeVD to explore the hemodynamic mechanisms underlying symptom development, particularly the phenomena of distal interstitial overflow and decreased superficial venous flow.

**Fig 1:**
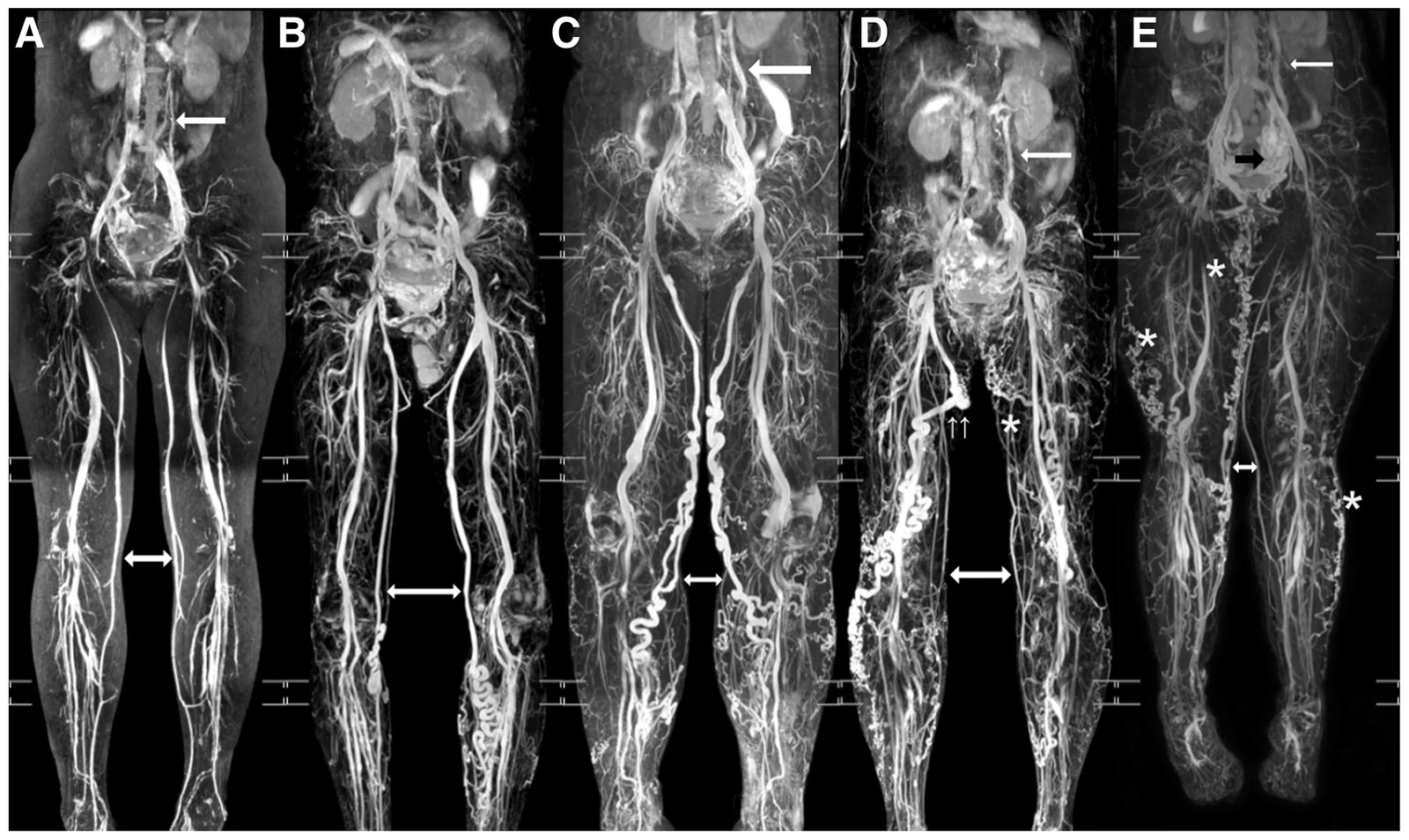
Lower-body venous map by two-dimensional phase-contrast magnetic resonance imaging (2D PC-MRI). **A,** Healthy female. **B,** Great saphenous vein (GSV, *double headed arrows*) reflux with varices in the calf (male). **C,** GSV truncal deformity with varices. An enlarged left ovarian vein is visible. **D,** Pelvic congestion with large left ovarian veins (*single headed arrow*), P-type pelvic leak (*left side,double upward arrow*) and vein of Giacomini-type accessory saphenous vein. **E,** Engorged bilateral ovarian veins, worm varix, and P-type and G-type pelvic leak (*asterisks*). Notably both GSVs are thin, compatible with decreased GSV outflow.

## Methods

### Participants

The Institutional Review Board (IRB) of Chang Gung Memorial Hospital approved this study (IRB Nos. 201700389B0, 201802137B0, 202001213B0, 202100938B0, 202200062B0, 202302156B0, and 202600425B0). This is a single institution, retrospective, observational cohort study. We collected complete hemodynamic data from 669 individuals who underwent NC-MRA for the evaluation of their lower body at a tertiary hospital between April 2017 and February 2024. All data were prospectively collected and retrospectively analyzed after IRB approvement. Male were not included in this study. We excluded patients who were pregnant, had restless leg syndrome, arrhythmia, morbidly obese, and MRI-incompatible devices. Initially, the 241 male participants were excluded. Of the remaining female participants were classified into the PeVD group if they presented with characteristic pelvic symptoms (eg, CPP, dyspareunia, vulvar/genital varicosities, and venous claudication) accompanied by echogram/2D PC-MRV evidence of pelvic venous pathology [left gonadal vein (LGV) diameter > 8 mm with retrograde flow/reflux and/or dilated pelvic venous plexuses > 5 mm]. Patients presenting with lower-extremity or abdominal/pelvic complaints who lacked these predefined anatomic and hemodynamic pelvic venous criteria were classified into the Symptomatic Non-PeVD group. The 39 healthy controls (HCs) were also included among these 428 participants.

The cohort consisted of 428 participants: 138 (32.24%) with PeVD, 251 (58.65%) with non-PeVD, and 39 (9.11%) HCs. Among the 138 patients diagnosed with PeVD, 26 patients received gonadal intervention because of inadequate relief of discomfort through conservative methods (lifestyle modification and pharmacotherapy). All patients with PeVD were evaluated by a gynecologist for potential malignancies. Before a scheduled MRI was conducted, an US was performed with the patient in the supine position. The venous condition of the 39 HCs was examined through MRI. Adult women without any known vascular disease of the lower extremities were included as HCs. Fig 2 presents a flowchart of patient identification and exclusion for the study cohort.

**Fig 2:**
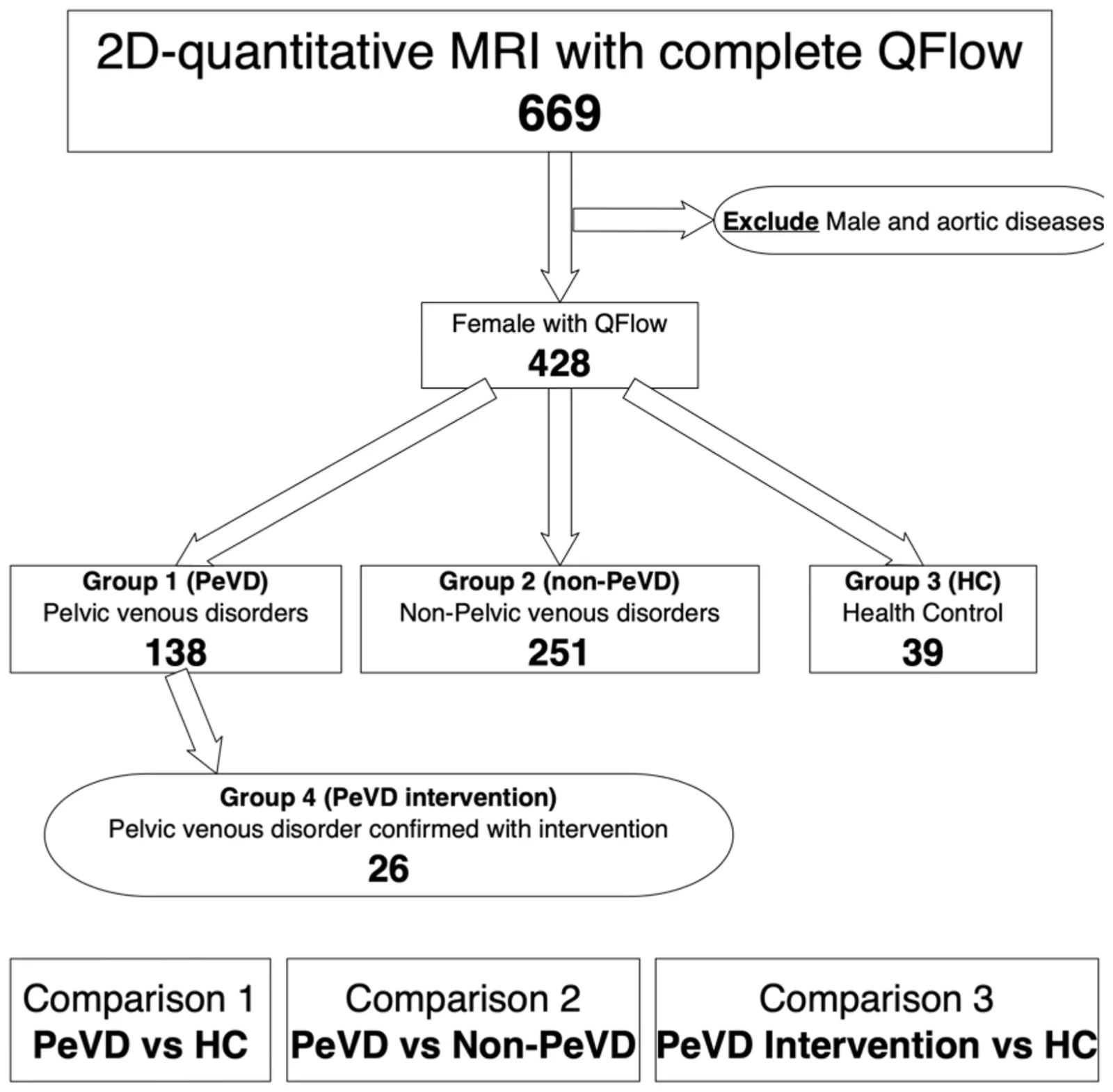
Flow chart of the study cohort. *2D*, Two-dimensional; *HC*, health control; *MRI*, magnetic resonance imaging; *PeVD*, pelvic venous disorders.

The 138 patients with PeVD had relevant symptoms and signs, including pelvic venous pain, dyspareunia, discomfort or heaviness in the hypogastric region, and vulvar varicose veins. Quantitative 2D PC-MRI demonstrated reflux in the dilated LGV (>8 mm in diameter). In addition, characteristic pelvic varicosities were observed within the parauterine, paraovarian, and myometrial venous plexuses, presenting as tortuous veins measuring >5 mm in diameter.^22^^,^^23^ In addition, we recorded information on the patients' demographics, Symptoms-Varices-Pathophysiology (SVP) classification, venous characteristics, medications, and interventions for venous diseases.^4^^,^^24^^,^^25^ This study was prospectively registered in the Research Registry (UIN: researchregistry10167) and reported in line with the Strengthening the Reporting of Cohort, Cross-sectional and Survey Studies criteria.^26^

### MRI data acquisition

The examination was performed on a 1.5 Tesla Philips Ingenia MRI scanner with electrocardiogram gating. Image and data analyses were executed using its integrated software (Ingenia Rev version 4 rev.01; Philips Medical Systems Nederland B.V.). The protocol included gated 3D TSE-STIR MRV for anatomy and 2D PC-MRI for hemodynamics. Systolic triggering and STIR suppression produced venous-only 3D datasets. MRV was acquired at four coronal levels using standard TSE-STIR parameters. Quantitative 2D PC-MRI was obtained at four axial planes (TR, 16 ms; TE, 8 ms; slice, 5 mm; pixel, 0.33 mm; VENC, 80 cm/s). The entire MRI process—including image acquisition, image reconstruction, and 2D PC-MRI analysis—required 25 minutes.^16^^,^^20^

### Hemodynamic variables

The measurements and analysis were performed by a designated radiologic senior technologist (S.C.W.) and were then jointly confirmed by a vascular surgeon (Y.K.H.) and a radiologist (C.W.C.). ROI were manually drawn on axial images of the IVC, external iliac vein (EIV), femoral vein (FV), popliteal vein (PV), GSV, and LGV to encompass the full lumen (Fig 3, *A* and *B*). Phase-shift data within each ROI were used to compute flow parameters. The volumetric variables [eg, stroke volume (SV)] were calculated by temporally integrating the cross-sectional lumen area (A) and the mean velocity (MV; v) across the cardiac cycle, using the standard formula: V = ∫[A(t) × v(t)] dt. Although the spatial acquisition is 2D, the high temporal resolution allows for precise quantification of dynamic volumetric transport within the target vessel (ROI). We used Philips QFlow for its optimized automated edge-detection workflow.

**Fig 3:**
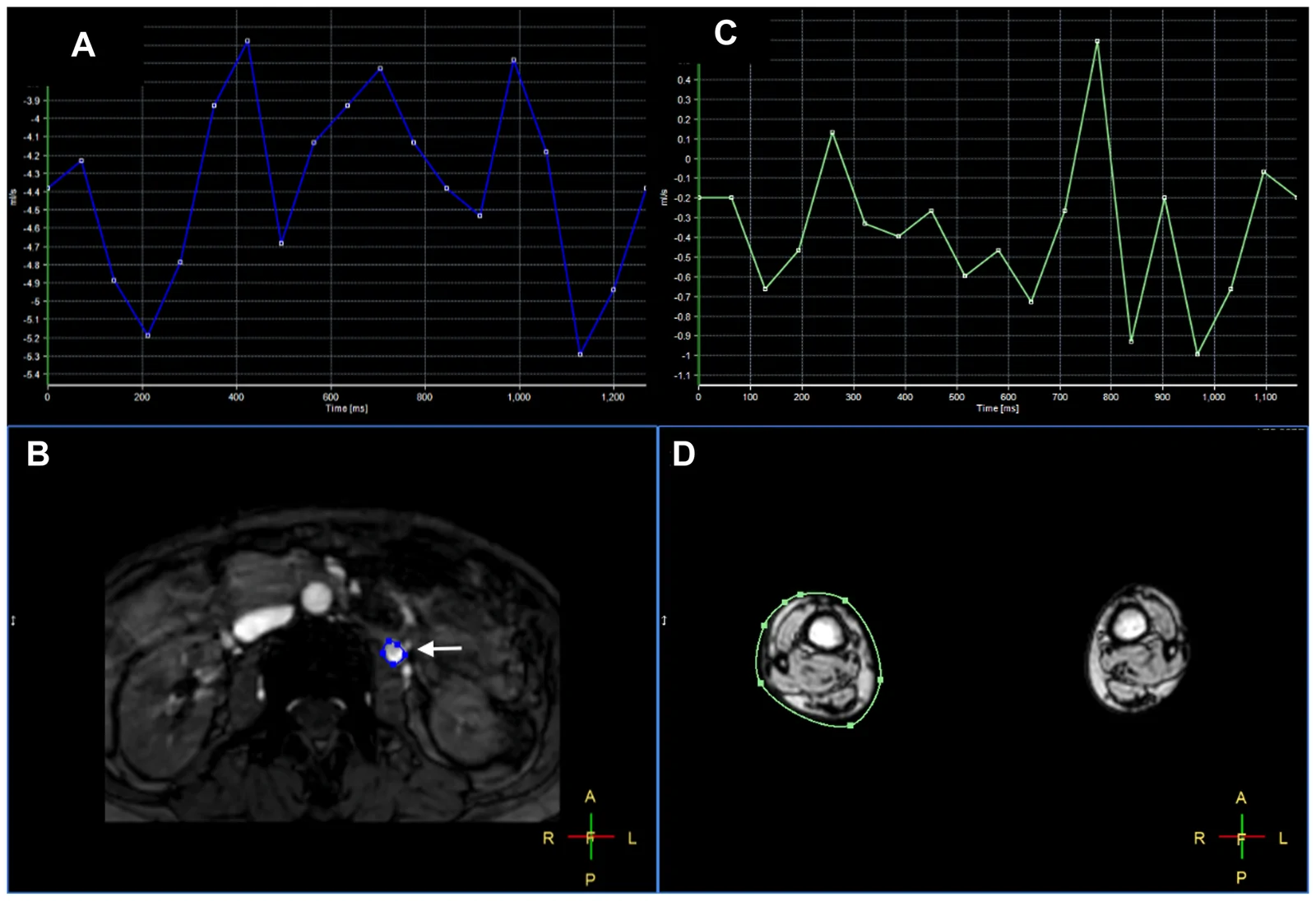
Example of QFlow analysis. **A,** QFlow parameter though selected regions of interest (ROI) as left ovarian veins. **B,** Selected left ovarian vein. **C,** Regional QFlow at calves. **D,** Selected area for regional QFlow analysis.

Additional ROIs covering the entire calf cross-section generated right and left regional calf signals after removing vessel pixels (Fig 3, *C* and *D*). The magnetic phase shift measured within this area reflects the interstitial fluid status of the surrounding tissues and serves as an indirect indicator of tissue perfusion around the ankle (ankle perfusion). Seven QFlow variables were obtained: SV, forward flow volume (FFV), backward flow volume (BFV), absolute stroke volume (ASV), mean flux (MF), stroke distance (SD), and MV, each representing volumetric or velocity-derived hemodynamic measures. Flow volume elevation indicates increased SV and ASV. All patients undergo heart rate monitoring and calibration. The device continuously tracks heart rate during scans, automatically adjusting signal acquisition. QFlow software requires no body mass index, body surface area, or blood pressure calibration.

### Statistical analysis

Demographic characteristics among the three groups (PeVD, non-PeVD, and HC) were compared using one-way analysis of variance for continuous variables and Fisher exact test for categorical variables. Agreement between venography and 2D PC-MRI in the LGV was assessed using Cohen kappa. Because flow variables were non-normally distributed, they are presented as median (interquartile range) and were compared using the Kruskal-Wallis test, followed by pairwise comparisons with Bonferroni correction. Diagnostic discrimination (PeVD vs HC; PeVD vs non-PeVD) was evaluated via receiver-operating characteristic analysis, with optimal cut-offs derived from the Youden index. A two-tailed *P* value <.05 was considered statistically significant. All statistical analyses were performed using SPSS version 25.0 (IBM Corp.).

## Results

### Participant characteristics

Table I lists the demographic characteristics of the 138 female patients with PeVD, including age, comorbidities, relevant symptoms and signs (SVP classification), venous morphology on MRI, visual analog scale scores, medication, and venous intervention. Most of the patients experienced CPP, along with extrapelvic symptoms such as dyspareunia, genital discomfort, nonsaphenous thigh pain, sciatica, and venous claudication. Venous morphology was predominantly characterized by pelvic varices, including some varicose veins over the genital area and pelvic escape points. Notably, 67 of the 138 patients (48.5%) exhibited caudal flow in the LGV (left ovarian vein) and 57 (41.3%) had a bigger LGV, as confirmed by quantitative 2D PC-MRI. More than half of the patients with PeVD received micronized purified flavonoid fraction, pentoxifylline, and nonsteroidal anti-inflammatory drugs. Furthermore, 26 patients received gonadal intervention with embolization for the LGV or sclerotherapy for LVG branches, 26 patients received GSV truncal ablation plus sclerotherapy, and one patient received angioplasty for her left EIV.

**Table I:** Demographic data of patients with pelvic venous disorders (*PeVD*), without PeVD, and health control (*HC*)

Variables	PeVD	Non-PeVD	HC
Total N = 428 (100%)	N = 138 (32.3)	N = 251 (58.6)	N = 39 (9.1)
Age, years	56.83 ± 14.52	64.06 ± 14.40	36.25 ± 14.24
Comorbidities			
Smoking	2 (1.4)	2 (0.8)	0
Hypertension	21 (15.2)	2 (0.8)	0
Diabetes	8 (5.8)	0	0
Clinical manifestation			
Back pain (SVP, S1)	8 (5.8)	1 (0.4)	0
Chronic pelvic pain (SVP S2)	126 (91.3)	2 (0.8)	0
Extrapelvic symptoms of venous origin (SVP S3)	93 (67.4)	87 (34.7)	0
Dyspareunia, genital pain, and ovarian tenderness (SVP S3a)	39 (28.3)	0	0
Posteromedial thigh (nonsaphenous) pain and sciatica (SVP S3b)	38 (27.5)	0	0
Venous claudication (SVP S3c)	28 (20.3)	87 (34.7)	0
VAS	3.89 ± 1.24	1.28 ± 1.04	0
Venous morphology			
Renal hilar varices (SVP V1)	8 (5.8)	0	0
Pelvic varices (SVP V2)	119 (86.2)	15 (6)	0
Pelvic origin extrapelvic varices (SVP V3)	72 (52.2)	0	0
Genital varices (SVP V3a)	60 (43.5)	0	0
Pelvic origin varicose vein from pelvic escape points (SVP V3b)	40 (29)	0	0
Pathophysiology			
Obstruction	15 (10.9)	4	0
Reflux	123 (89.1)	120 (47.8)	0
Etiology			
Nonthrombotic	138 (100)	212 (84.5)	0
MRI figures for veins			
LGV diameter over 4 mm	86 (62.3)	34 (13.5)	2 (5.1)
LGV diameter over 8 mm	57 (41.3)	3 (1.2)	0
LGV caudal flow	67 (48.6)	11 (4.4)	0
Uterine varices over 5 mm	86 (62.3)	10 (4.0)	0
Vulva and genital varices	45 (32.6)	15 (6)	0
GSV varices	46 (33.3)	137 (54.6)	0
Lateral thigh varices	42 (30.4)	71 (28.3)	0
Medication			
Micronized purified flavonoid fraction	66 (47.8)	45 (17.9)	0
Pentoxifylline	74 (53.6)	86 (34.3)	0
Clonazepam	12 (8.7)	37 (14.7)	0
NSAID	66 (47.8)	40 (15.9)	3 (7.7)
Intervention			
LGV embolization	26 (18.8)	0	0
Sclerotherapy (Fibrovein)	21 (15.2)	30 (12.0)	0
GSV ablation	26 (18.8)	99 (39.4)	0
Angioplasty plus stent	1 (0.7)	2 (0.8)	0

*GSV*, Greater saphenous vein; *LGV*, left gonadal vein; *MRI*, magnetic resonance imaging; *NSAID*, nonsteroidal anti-inflammatory drug; *SVP*, Symptoms-Varices-Pathophysiology classification; *VAS*, visual analog scale.

Categorical data are shown as number (%).

### 2D PC-MRI QFlow in PeVD and HCs

The parameters of the 138 patients with PeVD significantly differed from those of the 39 HCs (Table II). 2D PC-MRI QFlow revealed statistically significant differences in several venous segments:

**Table II:** The comparison of QFlow parameters between pelvic venous disorders (PeVD), non-PeVD, and Health control patients

QFlow parameters	PeVD (N = 138)	Non-PeVD (N = 251)	Health control (N = 39)	*P* value (a<.05)	*ANOVA P* value^a^
Stroke volume (SV), mL					
IVC	16.188 (15.302-17.072)	13.373 (12.510-14.244)	15.582 (13.662-17.501)	.0001^a^/.5375	.0005^a^
Right PV	0.7291 (0.6443-0.8140)	0.8822 (0.7951-0.9693)	0.5690 (0.4864-0.6942)	.0134^a^/.0139^a^	.0285^a^
Left PV	0.7199 (0.6434-0.7964)	0.8677 (0.7767-0.9587)	0.5610 (0.4809-0.6699)	.0147^a^/.0068^a^	.0380^a^
LGV	1.2870 (1.0418-1.5323)	0.5400 (0.3488-0.7312)	0.5362 (0.4200-0.7154)	.0001^a^/.0001^a^	.0002^a^
RG	0.2444 (0.1591-0.3297)	0.1229 (0.0946-0.1511)	0.0890 (0.0470-0.1513)	.0082^a^/.0018^a^	.0167^a^
LG	0.2554 (0.1782-0.3327)	0.1157 (0.0853-0.1461)	0.1246 (0.0650-0.2127)	.0011^a^/.0118^a^	.0023^a^
Right EIV	4.6454 (4.3260-4.9746)	4.9395 (4.6246-52545)	3.8736 (3.3985-4.4658)	.2373/.0089^a^	.2032
Left EIV	4.2069 (3.9178-4.4997)	4.3297 (4.0437-4.6158)	3.6423 (3.1671-4.2049)	.5826/.0422^a^	.5496
Right GSV	0.2355 (0.1770-0.2911)	0.2850 (0.2408-0.3292)	0.3546 (0.2923-0.4626)	.1819/.0182^a^	.5016
Left GSV	0.2474 (0.1810-0.3242)	0.3194 (0.2544-0.3843)	0.3933 (0.3172-0.5182)	.1630/.0161^a^	.4778
Foreword flow volume (FFV), mL					
IVC	16.717 (15.808-17.627)	13.691 (12.819-14.581)	16.186 (14.243-18.130)	.0001^a^/.5976	.0002^a^
Right PV	0.7359 (0.6519-0.8322)	0.8929 (0.8004-0.9727)	0.5849 (0.5016-0.7121)	.0107^a^/.0206^a^	.0253^a^
Left PV	0.7150 (0.6383-0.7994)	0.8647 (0.7730-0.9497)	0.5659 (0.4875-0.6736)	.0141^a^/.0104^a^	.0396^a^
RG	0.2021 (0.1209-0.2985)	0.1020 (0.0751-0.1264)	0.0697 (0.0295-0.1259)	.0286^a^/.0185^a^	.0557
LG	0.2193 (0.1456-0.3096)	0.1102 (0.0803-0.1375)	0.1167 (0.0565-0.2035)	.0114^a^/.0500^a^	.0239^a^
Right EIV	4.7198 (4.4028-5.0764)	5.0051 (4.6823-5.3278)	4.0362 (3.5692-4.6822)	.2603/.0264^a^	.2600
Right GSV	0.2425 (0.1831-0.2971)	0.2918 (0.2479-0.3357)	0.3644 (0.3011-0.4664)	.1815/.0128^a^	.4968
Left GSV	0.2527 (0.1862-0.3293)	0.3106 (0.2572-0.3640)	0.3956 (0.3179-0.5187)	.1970/.0176^a^	.5215
LGV	0.3162 (0.2463-0.3985)	0.3000 (0.1485-0.4515)	0.5097 (0.3752-0.6911)	.8800/.0185^a^	.8325
Mean flux (MF), mL/min					
IVC	18.517 (17.516-19.711)	15.547 (14.520-16.642)	17.908 (15.511-20.305)	.0002^a^/.6108	.0050^a^
Left FV	1.4707 (1.3355-1.6354)	1.6839 (1.5372-1.8060)	1.3472 (1.1023-1.5920)	.0354^a^/.4222	.1005
Right PV	0.8138 (0.7244-0.9276)	1.0091 (0.9029-1.1009)	0.6092 (0.5145-0.7501)	.0063^a^/.0061^a^	.0127^a^
Left PV	0.8018 (0.7176-0.9012)	0.9886 (0.8793-1.0939)	0.6064 (0.5099-0.7335)	.0072^a^/.0045^a^	.0189^a^
LGV	1.4953 (1.1641-1.7466)	0.6217 (0.4198-0.8212)	0.6028 (0.4691-0.8052)	.0001^a^/.0001^a^	<.0001^a^
RG	0.2736 (0.1797-0.3881)	0.1370 (0.1045-0.1662)	0.1041 (0.0540-0.1780)	.0114^a^/.0040^a^	.0212^a^
LG	0.2823 (0.2029-0.3829)	0.1325 (0.0962-0.1657)	0.1431 (0.0748-0.2440)	.0019^a^/.0182^a^	.0045^a^
Right EIV	5.2183 (4.8346-5.6754)	5.6542 (5.2793-6.0292)	4.1913 (3.6529-4.8980)	.1458/.0035^a^	.1087
Left EIV	4.6894 (4.3612-5.0510)	4.9699 (4.6232-5.3167)	3.9503 (3.3546-4.6202)	.2978/.0283^a^	.2908
Right GSV	0.2617 (0.1957-0.3270)	0.3261 (0.2741-0.3781)	0.3936 (0.3185-0.5147)	.1367/.0227^a^	.9207
Left GSV	0.2793 (0.2071-0.3639)	0.3567 (0.2890-0.4244)	0.4269 (0.3395-0.5594)	.1552/.0270^a^	.9326
Stroke distance (SD), cm/s					
IVC	9.7966 (9.2045-10.576)	8.2590 (7.6176-8.8256)	10.774 (8.9096-12.638)	.0009^a^/.2238	.0047^a^
Right PV	1.2380 (1.1266-1.3713)	1.5011 (1.3563-1.6231)	1.0395 (0.9386-1.2174)	.0040^a^/.0280^a^	.0150^a^
LGV	−0.4998 (−1.1497 to 0.1501)	0.3651 (0.0111-0.7193)	2.1385 (1.3696-2.9074)	.0219^a^/.0001^a^	.0714
Left FV	2.9979 (2.7133-3.2879)	3.3344 (3.0665-3.6023)	4.0318 (3.1021-5.2322)	.1154/.0432^a^	.3935
Right GSV	1.0921 (0.8512-1.3084)	1.2878 (1.0948-1.4809)	2.0997 (1.6858-2.5907)	.2173/.0004^a^	.3120
Left GSV	1.0775 (0.8652-1.3457)	1.3840 (1.1181-1.6499)	2.3200 (1.9091-3.0183)	.1303/.0001^a^	.2843
Mean velocity (MV), cm/s					
IVC	11.280 (10.598-12.246)	9.5778 (8.8476-10.218)	12.449 (10.336-14.562)	.0017^a^/.2174	.0064^a^
Right FV	3.1665 (2.8815-3.5303)	3.6519 (3.3419-3.9023)	3.9303 (3.0066-4.8540)	.0240/.0489^a^	.2165
Left FV	3.3529 (3.0365-3.6986)	3.8223 (3.4778-4.1054)	4.4413 (3.2681-5.6144)	.0433^a^/.0141^a^	.3167
Right GSV	1.2203 (0.9428-1.4823)	1.4516 (1.2327-1.6705)	2.3156 (1.8214-2.8135)	.2019/.0009^a^	.3368
Left GSV	1.2214 (0.9736-1.5342)	1.5852 (1.2739-1.8576)	2.5279 (2.0547-3.2522)	.0744/.0002^a^	.6088
Right PV	1.3880 (1.2569-1.5498)	1.7249 (1.5551-1.8680)	1.1136 (0.9901-1.3202)	.0018^a^/.0101^a^	.0065^a^
LGV	−0.4880 (−1.2361 to 0.2602)	0.3953 (−0.016 to 0.8068)	2.3869 (1.5126-3.2511)	.0422^a^/.0001^a^	.1138

*ANOVA*, Analysis of variance; *EIV*, external iliac vein; *FV*, femoral vein; *GSV*, great saphenous vein; *IVC*, inferior vena cava; *LG*, left calf region; *LGV*, left gonadal vein; *PV*, popliteal vein; *RG*, right calf region.

a Statistically significant (*P* < .05).

In the *iliac veins*, both SV and MF were significantly higher in the right and left EIVs of the PeVD group (eg, SV in the right EIV: 4.65 vs 3.87 mL, *P* = .0089; MF in the right EIV: 5.22 vs 4.19 cm^3^/s; *P* = .0035), reflecting increased upstream venous recirculation or redistribution of flow toward the iliac system. In contrast, the great saphenous veins (GSV) demonstrated significantly lower SV, FFV, MF, and SD values in patients with PeVD compared with controls on both sides (eg, SV in the left GSV: 0.25 vs 0.39 mL; *P* = .0161; MF in the left GSV: 0.28 vs 0.43 cm^3^/s; *P* = .0270), indicating reduced effective outflow through superficial venous pathways.

A particularly finding was observed in the LGV. Patients with PeVD exhibited markedly elevated SV (1.29 vs 0.54 mL; *P* = .0001), MF (1.50 vs 0.60 cm^3^/s; *P* = .0001), and ASV (1.36 vs 0.57 mL; *P* = .0001), accompanied by strongly negative SD and MV values (SD in LGV: −0.50 vs 2.14, *P* = .0001; MV in LGV: −0.49 vs 2.39, *P* = .0001). This reflects dominant *retrograde flow through the gonadal vein*, consistent with the classical pathophysiology of PeVD. These LGV findings showed the largest effect sizes among all segments, underscoring their diagnostic significance. FVs displayed less consistent differences, but the left FV showed a higher cross-sectional area (0.30 vs 0.21 cm^2^; *P* = .0379) and a lower SD/MV in patients with PeVD, suggesting relative stagnation or altered compliance.

PVs also exhibited consistently higher SV, FFV, and MF in patients with PeVD bilaterally (eg, MF in the left PV: 0.80 vs 0.61 cm^3^/s; *P* = .0045), suggesting a higher venous burden under elevated pelvic venous pressures. Finally, *significant reductions in flow pulsatility and peak velocities in the GSV and FVs* were observed (eg, SD in the left GSV: 1.08 vs 2.32, *P* = .0001; MV in the left GSV: 1.22 vs 2.53, *P* = .0002), further indicating impaired superficial venous dynamics in PeVD.

ROC analysis identified several *segments with moderate discriminative ability* for PeVD. Notably, *GSV parameters achieved the highest area under the curve (AUC) values* (right GSV SV: AUC, 0.689; left GSV SV: AUC, 0.706; both *P* < .0001), followed by LGV SD (AUC, 0.714; *P* < .0001) and MV (AUC, 0.703; *P* < .0001). These results indicate that *retrograde LGV flow and reduced GSV flow (hypoflow) are the most sensitive hemodynamic markers* for differentiating PeVD from HCs (Supplementary Table I, online only).

Fig 4 demonstrated the hierarchical clustering heatmap of QFlow values comparing PeVD and HCs. Columns represent venous segments, and rows indicate flow parameters (area, SV, MF, SD, and MV). LGV-dominant cluster reflects pelvic venous reflux signature, whereas calf area clusters reflect distal interstitial overflow and compensatory decreased flow of superficial veins (GSV hypoflow).

**Fig 4:**
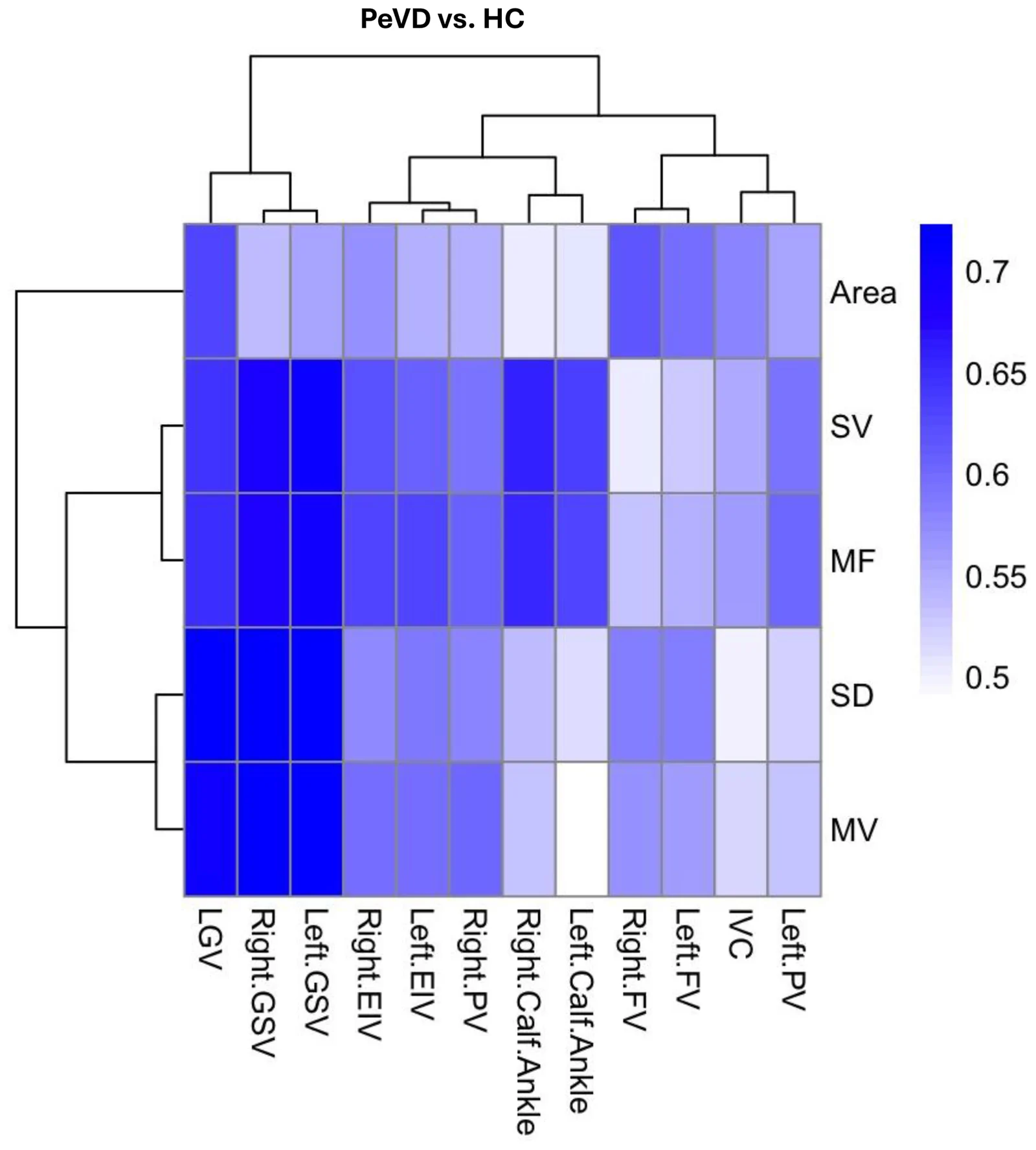
Hierarchical clustering heatmap of QFlow values comparing pelvic venous disorders (*PeVD*) and healthy control (*HC*); PeVD vs non-PeVD. *EIV*, External iliac vein; *FV*, femoral vein; *GSV*, great saphenous vein; *IVC*, inferior vena cava; *LGV*, left gonadal vein; *MF*, median frequency; *MV*, median velocity; *PV*, popliteal vein; *SD*, standard deviation; *SV*, stroke volume.

### 2D PC-MRI QFlow analysis of PeVD and non-PeVD

Table II also lists the findings of the 2D PC-MRI hemodynamic analysis for 138 patients with PeVD and 251 non-PeVD. QFlow analysis revealed distinct hemodynamic features between PeVD and non-PeVD groups. *IVC flow parameters were consistently higher*, for example SV in IVC was 16.2 mL in PeVD vs 13.4 mL in non-PeVD (*P* = .0001), indicating augmented central venous return. *PVs showed lower flow*, such as MF in the right PV (0.81 vs 1.01 cm^3^/s; *P* = .0063), reflecting reduced deep venous outflow and stasis. *LGV parameters were striking*, with negative SD in LGV (−0.50 vs 0.37; *P* = .0219) indicating dominant reflux along the gonadal axis.

LGV flow-volume analysis provided the strongest discriminatory capability (AUC, ∼0.76), surpassing reflux-velocity markers. Calf venous signals (AUC, ∼0.66) demonstrated distal ankle interstitial overflow, whereas IVC flow volume elevation (AUC, ∼0.60) supported a central augmentation phenomenon. These findings establish PeVD as a multisegmented hemodynamic disorder characterized by pelvic reservoir failure with propagated downstream venous hypertension (Supplementary Table II, online only). LGV AUC clustering revealed the strongest discriminatory capacity among all venous segments, followed by calf venous signals, supporting a three-level PeVD physiology: central flow enhancement, pelvic venous stasis and distal interstitial overflow (Fig 5).

**Fig 5:**
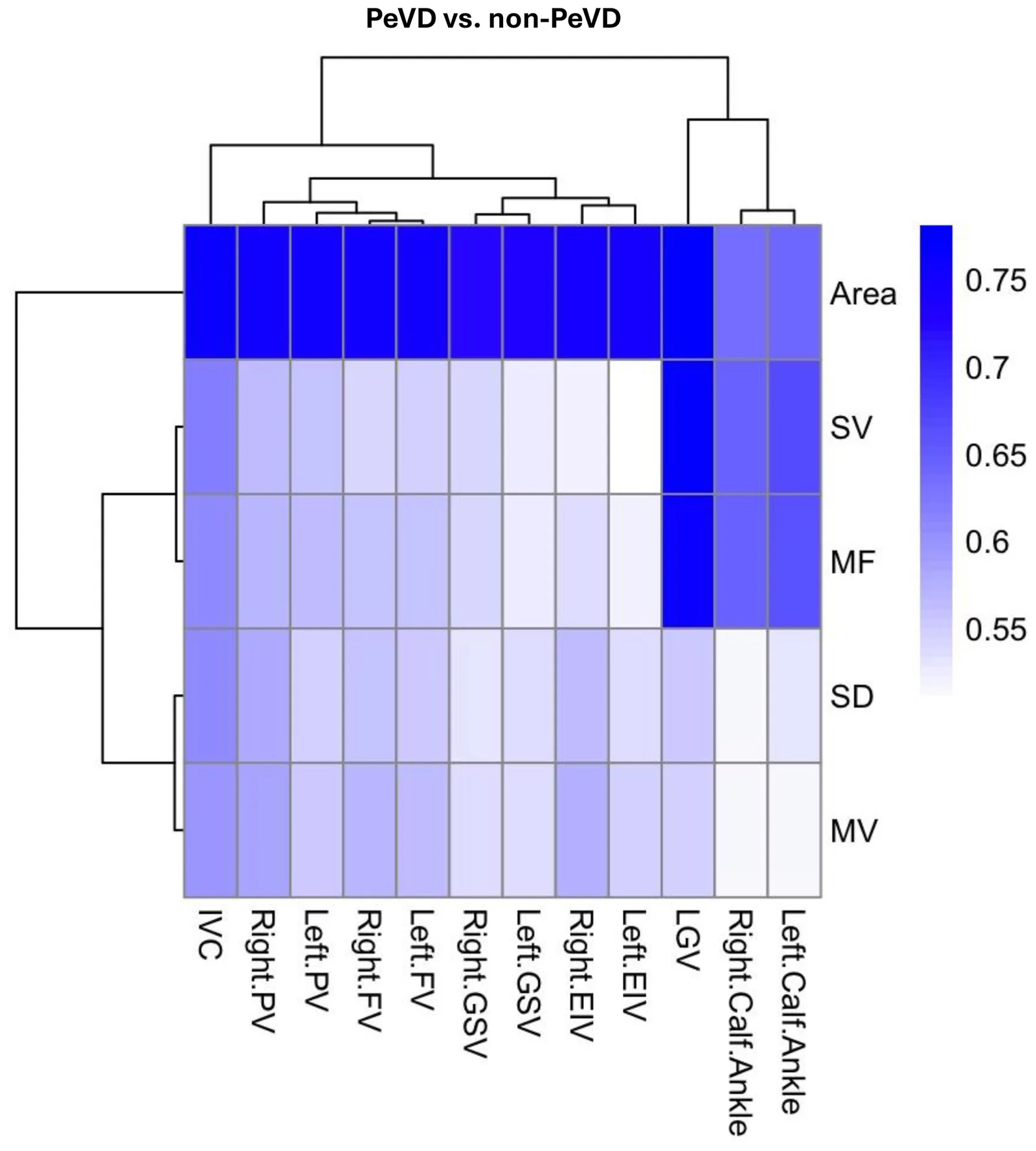
Hierarchical clustering heatmap of QFlow values comparing patients with pelvic venous disorders (*PeVD*) and without PeVD. *EIV*, External iliac vein; *FV*, femoral vein; *GSV*, great saphenous vein; *IVC*, inferior vena cava; *LGV*, left gonadal vein; *MF*, median frequency; *MV*, median velocity; *PV*, popliteal vein; *SD*, standard deviation; *SV*, stroke volume.

### Symptomatic PeVD receiving gonadal intervention and control groups

Twenty-six patients underwent embolization of the LGV in accordance with the findings of preoperative 2D PC-MRI hemodynamic analysis and clinical symptoms. On venography, all 26 patients demonstrated gonadal vein reflux and tortuous uterine veins with diameters exceeding 5 mm. We compared their findings of 2D PC-MRI QFlow with health controls (Supplementary Table III, online only). Patients with symptomatic PeVD who underwent venous intervention showed a coherent hemodynamic signature vs HCs. *Superficial flow was reduced in GSV*, especially on the left (SV in the left GSV1: 0.15 vs 0.39 mL, *P* = .0002; MF in the left GSV1: 0.16 vs 0.43 cm^3^/s, *P* = .0002; SD/MV likewise lower on both sides, *P* ≤ .0004). *Gonadal reflux dominated*, with markedly higher LGV volumes and reversed directionality (SV in LGV: 1.99 vs 0.54 mL, *P* = .0007; MF in LGV: 2.40 vs 0.60 cm^3^/s, *P* = .0010; SD in LGV: −3.05 vs 2.14, *P* = .0001; MV in LGV: −3.67 vs 2.38, *P* = .0001). *Distal (ankle interstitial) signals were higher*, consistent with overflow (BFV in LG: 0.119 vs 0.026 cm^3^/s, *P* = .025; MF in RG: 0.22 vs 0.10 cm^3^/s, *P* = .047). Deep vein pulsatility was lower (eg, SD in the right FV: 2.43 vs 3.60; *P* = .0167). Diagnostic performance echoed these patterns: *GSV analysis* discriminated well (AUC, ∼0.69-0.72; eg, left GSV: SV [AUC, 0.706; *P* < .0001]; right GSV: MV [AUC, 0.715; *P* < .0001]), *LGV reflux indices* were similarly strong (SD: AUC, 0.714; MV AUC, 0.703; both *P* < .0001), whereas *ankle perfusion* was fair (AUC ∼0.63-0.66). Overall, *LGV reflux plus reduced GSV flow* are the most informative markers, with distal ankle (interstitial) signals providing supportive discrimination (Supplementary Table IV, online only). In particular, bilateral GSV and LGV parameters exhibited AUC values of approximately 0.70 with highly significant *P* values, supporting their discriminatory ability.

## Discussion

Because of the limited understanding of venous pathophysiology, predicting the outcomes of venous intervention remains challenging, with recurrence rates remaining high.^7^^,^^24^^,^^27^^,^^28^ For lower-body venous disorders, particularly those involving the pelvic venous system, objective diagnostic and hemodynamic tools were lacking before the adoption of 2D PC-MRI (Supplementary Video, online only). Routine clinical use of noninvasive 2DPC-MRI began at our institution in 2018, evaluation of more than 1000 subjects has revealed complex interactions within the pelvic venous network—including the gonadal, iliac, and renal veins—that influence superficial venous disease and treatment outcomes.^18^^,^^20^^,^^29^^,^^30^ Therefore, objective hemodynamic assessment may provide additional insights beyond anatomic imaging alone.

Our previous works have provided crucial hemodynamic evidence and objective morphological characterization of lower body venous disease, including pelvic leakage.^20^ “Pelvic leakage” refers to the pathophysiologic phenomenon in which elevated pelvic venous pressure escapes downward through pelvic floor pathways (eg, obturator, perineal, or gluteal escape points) into the lower extremities and may be associated with distal ankle interstitial signal shifts, reflecting increased interstitial fluid accumulation on 2D PC-MRI. In the present study, the 2D PC-MRI combined with QFlow analysis identified a reproducible hemodynamic signature of PeVD. Compared with HCs, PeVD showed increased IVC flow, suggesting augmented central venous return and possible recirculation at higher level from renal and hemiazygos system. The dominant caudal reflux in the LGV, characterized by negative SD and MV values, may contribute to this phenomenon. In addition, reduced forward flow in the PVs indicated attenuation of deep venous outflow, whereas diminished GSV flow and pulsatility, together with increased ankle interstitial signals (BFV/MF), were consistent with increased distal interstitial fluid accumulation and venous stasis. Similar directional differences were also observed when comparing patients with PeVD and symptomatic patients without PeVD. Together, these findings define a reproducible hemodynamic signature of PeVD. In contrast, superficial cutaneous venous drainage remains preserved through functionally competent GSV in supine, resulting in no compensatory increase in superficial venous flow or dilatation. This phenomenon may explain the observed superficial hypoflow, referred to as “compensatory decreasing flow of superficial vein.”

The coexistence of PeVD and lower-extremity venous disease has been increasingly recognized; therefore, these hemodynamic abnormalities have important implications for treatment planning. Professor Whiteley^28^^,^^31^ has reported that approximately one in six women presenting with lower-extremity varicose veins also have underlying PeVD. However, the recognition and appropriate management of PeVD have historically been hindered by the lack of validated definitions, randomized trials, and objective imaging criteria. The SVP classification has helped address these gaps by standardizing nomenclature and distinguishing the anatomic, hemodynamic, and etiologic domains.^4^ The management of PeVD or pelvic congestion syndrome should be staged and hemodynamics-guided.^8^ Conservative care, including activity modification (avoid prolonged standing/sitting), weight control, pelvic floor physiotherapy, compression, and pain education, offers low-risk symptom relief, especially for women of childbearing age and during pregnancy. Pharmacotherapy can stabilize flares but seldom corrects reflux/obstruction: nonsteroidal anti-inflammatory drugs; hormonal suppression (combined oral contraceptive pills or progestins; short gonadotropin-releasing hormone courses in refractory cases); and adjuncts such as venoactive agents and neuromodulators for CPP.^32^ Endovascular therapy targets the lesion and yields the most durable benefit when imaging confirms abnormalities.^3^^,^^12^^,^^24^^,^^33^ Consistent with our QFlow profile (LGV reflux with superficial venous hypoflow and supportive central/distal changes), selective embolization of refluxing gonadal/pelvic tributaries (coils/plugs ± sclerosants) is the first-line intervention. When significant iliac outflow obstruction coexists, iliac stenting can be considered with caution since the compression may be bilateral and multiple levels.^24^ Sclerotherapy for pelvic venous leak from either bottom-up or top-down way.^24^^,^^34^^,^^35^ Laparoscopic intervention including ovarian vein ligation have been reported with good result, but less favor in our institutions.^36^ 2D PC-MRI/QFlow aids selection, documents early response, supports investigation, and is helpful to explain the interventional plan with the patients. Gonadal vein embolizations or surgical ligations are safe and reduce pelvic pain in appropriately selected patients with PeVD. In this study, there were 26 patients received gonadal vein embolization. In discrimination testing, LGV reflux metrics (eg, SD/MV) yielded the best AUCs in surgery-confirmed PeVD vs healthy (AUC, ∼0.88), with GSV flow next (AUC, ∼0.69-0.77), whereas central and distal measures provided moderate but supportive separation. These observations support a lesion-directed strategy (reflux and/or obstruction) guided by objective hemodynamics. Although current clinical guidelines have integrated MRI into the management algorithm for PeVD, these protocols typically rely on time-of-flight sequences or CE-MRI rather than the 2D PC-MRI used in our study.^5^^,^^37^ Nevertheless, 2D PC-MRI is ideal for young women of childbearing age, facilitating longitudinal assessments of immediate postintervention results and potential recurrence without radiation or contrast. This valuable technique is expected to be widely adopted by venous specialists soon.

### Limitations

This study is retrospective and nonrandomized, with a smaller, younger, all-female control group. Another limitation of this study is that all MRI examinations were performed in the supine position. Consequently, posture-dependent and ambulation-related changes in venous hemodynamics could not be assessed. Future work should include larger prospective cohorts, longer follow-up, and alignment with emerging core outcome sets to correlate QFlow signatures with patient-reported outcomes and recurrence.

## Conclusions

Quantitative 2D PC-MRI provides an objective hemodynamic profile of PeVD. LGV reflux and superficial outflow attenuation emerged as key signatures. Integrating QFlow into selection and follow-up may improve diagnostic confidence and enable personalized, hemodynamics-guided venous interventions in line with contemporary practice.

## Author Contributions

Conception and design: SW, CLi, YCH, TY, CWC, YH

Analysis and interpretation: SW, CLi, CCK, YCH, SC, YLa, YLi, MW, YH

Data collection: SW, CLi, CCK, YCH, CLe, SC, YLa, YLi, MW, CWC, YH

Writing the article: SW, CCK, YCHs, SC, YLa, YLi, MW, YHu

Critical revision of the article: CLi, YCHs, CLe, TY, CWC, YHu

Final approval of the article: SW, CLi, CCK, YCH, CLe, TY, SC, YLa, YLi, MW, CWC, YH

Statistical analysis: CLi, YCH

Obtained funding: MW, CWC, YH

Overall responsibility: YH

C Li and S W contributed equally as first authors to this work

C C and YHu share senior authorship and co-corresponding this work

## Disclosures

None.

## Appendix (online only)

Supplementary Table I

Supplementary Table II

Supplementary Table III

Supplementary Table IV

Supplementary Video This video provides a detailed diagnostic comparison of female pelvic venous anatomy using two-dimensional phase-contrast magnetic resonance imaging (2D PC-MRI). It illustrates the differences between a healthy venous system, and various pathological conditions included gonadal venous reflux, iliac vein reflex, and compression.
