# Supplementary material for: Pelvic venous disorders: Objective hemodynamic signature from quantitative two-dimensional phase-contrast magnetic resonance imaging
Source: J Vasc Surg Venous Lymphat Disord. 2026 Sep 5;15(1):102616. doi: 10.1016/j.jvsv.2026.102616 (PMC13673354; doi:10.1016/j.jvsv.2026.102616)
Supplement: Supplementary file 2 [file mmc2.docx]

| Table S2: ROC for pelvic venous disorders (PeVD, n = 138) and NPCS (n = 251) by each venous segments and calves’ regions: ROC discrimination test | | | | |
| --- | --- | --- | --- | --- |
| Parameters | OR (95% CI) | P-value | AUC (95% CI) | P-value* |
| SV: Stroke Volume (mL) | | | | |
| IVC | 1.072 (1.036 1.110) | 0.0001 | 0.6208 (0.5645 0.6770) | 0.0001 |
| Right PV | 0.647 (0.441 0.950) | 0.0264 | 0.5662 (0.5083 0.6241) | 0.0251 |
| Left PV | 0.660 (0.449 0.971) | 0.0349 | 0.5578 (0.4995 0.6161) | 0.0520 |
| LGV | 1.504 (1.244 1.819) | 0.0001 | 0.7654 (0.7187 0.8120) | 0.0001 |
| Right Calf ankle | 4.320 (1.792 10.41) | 0.0011 | 0.6470 (0.5943 0.6998) | < 0.0001 |
| Left Calf ankle | 4.457 (1.938 10.25) | 0.0004 | 0.6670 (0.6145 0.7195) | < 0.0001 |
| MF: Mean Flux (mL/min) | | | | |
| IVC | 1.048 (1.019 1.077) | 0.0009 | 0.6082 (0.5518 0.6646) | 0.0002 |
| Right PV | 0.657 (0.470 0.919) | 0.0141 | 0.5719 (0.5142 0.6296) | 0.0146 |
| Left PV | 0.670 (0.479 0.938) | 0.0197 | 0.5642 (0.5062 0.6222) | 0.0301 |
| LGV | 1.431 (1.217 1.681) | 0.0001 | 0.7621 (0.7151 0.8090) | 0.0001 |
| Right Calf Region | 3.718 (1.675 8.255) | 0.0012 | 0.6484 (0.5957 0.7011) | < 0.0001 |
| Left Calf Region | 3.383 (1.640 6.980) | 0.0010 | 0.6655 (0.6129 0.7180) | < 0.0001 |
| SD: Stroke Distance (cm/s) | | | | |
| IVC | 1.075 (1.027 1.125) | 0.0021 | 0.6086 (0.5523 0.6648) | 0.0002 |
| Right PV | 0.737 (0.581 0.934) | 0.0118 | 0.5798 (0.5224 0.6371) | 0.0064 |
| MV: Mean Velocity (cm/s) | | | | |
| IVC | 1.062 (1.021 1.105) | 0.0030 | 0.5950 (0.5380 0.6519) | 0.0011 |
| Right FV | 0.889 (0.798 0.992) | 0.0348 | 0.5690 (0.5097 0.6283) | 0.0226 |
| Left FV | 0.911 (0.826 1.005) | 0.0628 | 0.5651 (0.5062 0.6241) | 0.0304 |
| Right PV | 0.753 (0.614 0.923) | 0.0063 | 0.5864 (0.5293 0.6436) | 0.0030 |
| Negative value (reflux) in Left gonadal vein | | | | |
| SD of LGV | 0.920 (0.861 0.983) | 0.0134 | 0.5553 (0.4883 0.6222) | 0.1059 |
| MV of LGV | 0.939 (0.887 0.993) | 0.0285 | 0.5501 (0.4832 0.6170) | 0.1420 |
