# Supplementary material for: Pelvic venous disorders: Objective hemodynamic signature from quantitative two-dimensional phase-contrast magnetic resonance imaging
Source: J Vasc Surg Venous Lymphat Disord. 2026 Sep 5;15(1):102616. doi: 10.1016/j.jvsv.2026.102616 (PMC13673354; doi:10.1016/j.jvsv.2026.102616)
Supplement: Supplementary file 3 [file mmc3.docx]

Table S3 The comparison between intervention-confirmed PeVD patients (PeVD op, n = 26) and health control patients (n = 39)

| No | Outcome | PeVD op (n = 26) | HC (n = 39) | P-value |
| --- | --- | --- | --- | --- |
| 6 | SV_3_Rt_GSV1 | 0.2123 (0.0779 0.3467) | 0.3546 (0.2733 0.4359) | 0.0700 |
| 7 | SV_3_Lt_GSV1 | 0.1504 (0.0717 0.2291) | 0.3933 (0.2962 0.4904) | 0.0002 |
| 12 | SV_1_LGV | 1.9850 (1.2143 2.7557) | 0.5362 (0.4007 0.6716) | 0.0007 |
| 13 | SV_5_RG_ankle | 0.2019 (0.1077 0.2962) | 0.0890 (0.0414 0.1366) | 0.0343 |
| 21 | FFV_3_Lt_GSV1 | 0.1569 (0.0793 0.2345) | 0.3956 (0.2994 0.4919) | 0.0002 |
| 26 | FFV_1_LGV | 0.1835 (0.0130 0.3539) | 0.5097 (0.3669 0.6526) | 0.0040 |
| 29 | BFV_2_Rt_EIV | 0.0054 (-0.0057 0.0165) | 0.1613 (0.0536 0.2689) | 0.0059 |
| 38 | BFV_1_LGV | 1.9177 (1.1301 2.7053) | 0.0579 (0.0199 0.0960) | 0.0001 |
| 40 | BFV_5_LG_ankle | 0.1185 (0.0416 0.1953) | 0.0259 (0.0012 0.0505) | 0.0250 |
| 42 | RF_2_Rt_EIV | 0.1177 (-0.1247 0.3601) | 3.4408 (1.3480 5.5335) | 0.0028 |
| 44 | RF_3_Rt_FV | 0.0450 (-0.0460 0.1360) | 1.7915 (0.3777 3.2054) | 0.0170 |
| 50 | RF_4_Lt_GSV2 | 16.173 (4.2017 28.144) | 3.3051 (0.2690 6.3413) | 0.0408 |
| 59 | MF_3_Rt_GSV1 | 0.2146 (0.0885 0.3407) | 0.3936 (0.2999 0.4873) | 0.0237 |
| 60 | MF_3_Lt_GSV1 | 0.1577 (0.0731 0.2423) | 0.4269 (0.3193 0.5345) | 0.0002 |
| 65 | MF_1_LGV | 2.4035 (1.4093 3.3976) | 0.6028 (0.4489 0.7567) | 0.0010 |
| 66 | MF_5_RG_ankle | 0.2204 (0.1186 0.3222) | 0.1041 (0.0476 0.1606) | 0.0470 |
| 71 | SD_3_Rt_FV | 2.4327 (1.8763 2.9891) | 3.5974 (2.8101 4.3848) | 0.0167 |
| 73 | SD_3_Rt_GSV1 | 0.9046 (0.4720 1.3373) | 2.0997 (1.6114 2.5881) | 0.0004 |
| 74 | SD_3_Lt_GSV1 | 0.8931 (0.3984 1.3878) | 2.3200 (1.7642 2.8758) | 0.0002 |
| 77 | SD_4_Rt_GSV2 | 0.9669 (0.4986 1.4353) | 1.0031 (0.7745 1.2317) | 0.0056 |
| 78 | SD_4_Lt_GSV2 | 0.7235 (0.4395 1.0074) | 1.1518 (0.9101 1.3935) | 0.0224 |
| 79 | SD_1_LGV | -3.0496 (-4.495 -1.604) | 2.1385 (1.3696 2.9074) | 0.0001 |
| 85 | MV_3_Rt_FV | 2.6831 (2.0662 3.3000) | 3.9303 (3.0066 4.8540) | 0.0258 |
| 87 | MV_3_Rt_GSV1 | 0.9296 (0.5027 1.3525) | 2.3156 (1.7446 2.8867) | 0.0002 |
| 88 | MV_3_Lt_GSV1 | 0.9346 (0.4050 1.4643) | 2.5279 (1.9135 3.1424) | 0.0002 |
| 92 | MV_4_Lt_GSV2 | 0.7815 (0.4697 1.0934) | 1.2223 (0.9690 1.4756) | 0.0289 |
| 93 | MV_1_LGV | -3.6650 (-5.425 -1.9048) | 2.3818 (1.5041 3.2595) | 0.0001 |
| 95 | MV_5_LG_ankle | -0.0007 (-0.0033 0.0017) | 0.0025 (0.0004 0.0046) | 0.0418 |
| 107 | Area_1_LGV | 0.2923 (0.1741 0.4105) | 0.1146 (0.0784 0.1508) | 0.0060 |
|  |  |  |  |  |
