# Supplementary material for: Pelvic venous disorders: Objective hemodynamic signature from quantitative two-dimensional phase-contrast magnetic resonance imaging
Source: J Vasc Surg Venous Lymphat Disord. 2026 Sep 5;15(1):102616. doi: 10.1016/j.jvsv.2026.102616 (PMC13673354; doi:10.1016/j.jvsv.2026.102616)
Supplement: Supplementary file 4 [file mmc4.docx]

| TableS4  ROC for intervention-confirmed PeVD (PeVD-op, n = 26) and health controls (n = 39) | | | | |
| --- | --- | --- | --- | --- |
| Parameters | OR (95% CI) | P-value | AUC (95% CI) | P-value* |
| SV: Stroke Volume (mL) | | | | |
| Right GSV | 0.368 (0.135 1.003) | 0.0506 | 0.6888 (0.6018 0.7757) | < 0.0001 |
| Left GSV | 0.441 (0.182 1.068) | 0.0697 | 0.7063 (0.6230 0.7897) | <0.0001 |
| Left PV | 2.973 (1.041 8.487) | 0.0418 | 0.5933 (0.4978 0.6887) | 0.0555 |
| LGV | 2.399 (1.296 4.442) | 0.0054 | 0.6462 (0.5593 0.7331) | 0.0010 |
| Right Calf ankle | 26.00 (2.522 268.2) | 0.0062 | 0.6569 (0.5695 0.7443) | 0.0004 |
| Left Calf ankle | 5.980 (0.982 36.39) | 0.0523 | 0.6344 (0.5398 0.7291) | 0.0054 |
| MF: Mean Flux (mL/min) | | | | |
| Right EIV | 1.258 (1.043 1.519) | 0.0166 | 0.6314 (0.5349 0.7278) | 0.0076 |
| Left EIV | 1.240 (1.009 1.525) | 0.0409 | 0.6307 (0.5327 0.7287) | 0.0087 |
| Right GSV | 0.431 (0.178 1.040) | 0.0612 | 0.6847 (0.5965 0.7729) | < 0.0001 |
| Left GSV | 0.505 (0.238 1.072) | 0.0752 | 0.6995 (0.6146 0.7843) | < 0.0001 |
| Right PV | 2.607 (1.045 6.501) | 0.0399 | 0.6065 (0.5116 0.7014) | 0.0279 |
| Left PV | 2.812 (1.089 7.261) | 0.0327 | 0.6039 (0.5080 0.6998) | 0.0338 |
| LGV | 2.184 (1.261 3.784) | 0.0053 | 0.6513 (0.5658 0.7369) | 0.0005 |
| Right Calf ankle | 15.29 (1.994 117.2) | 0.0087 | 0.6552 (0.5660 0.7445) | 0.0007 |
| Left Calf ankle | 4.463 (0.891 22.34) | 0.0687 | 0.6305 (0.5345 0.7266) | 0.0077 |
| SD: Stroke Distance (cm/s) | | | | |
| Right FV | 0.814 (0.677 0.979) | 0.0289 | 0.5853 (0.4789 0.6917) | 0.1162 |
| Left FV | 0.812 (0.693 0.952) | 0.0101 | 0.5818 (0.4779 0.6858) | 0.1228 |
| Right GSV | 0.653 (0.514 0.828) | 0.0004 | 0.7199 (0.6310 0.8088) | < 0.0001 |
| Left GSV | 0.616 (0.486 0.782) | < 0.0001 | 0.7234 (0.6301 0.8167) | < 0.0001 |
| LGV | 0.801 (0.711 0.902) | 0.0003 | 0.7140 (0.6322 0.7949) | < 0.0001 |
| MV: Mean Velocity (cm/s) | | | | |
| Left EIV | 1.064 (0.929 1.220) | 0.3708 | 0.6001 (0.5006 0.6996) | 0.0485 |
| Left FV | 0.854 (0.747 0.976) | 0.0205 | 0.5597 (0.4522 0.6673) | 0.2763 |
| Right GSV | 0.709 (0.576 0.873) | 0.0012 | 0.7145 (0.6248 0.8042) | < 0.0001 |
| Left GSV | 0.683 (0.560 0.832) | 0.0002 | 0.7152 (0.6226 0.8077) | < 0.0001 |
| Right PV | 1.770 (0.989 3.169) | 0.0544 | 0.6017 (0.5082 0.6953) | 0.0331 |
| LGV | 0.835 (0.754 0.924) | 0.0005 | 0.7031 (0.6220 0.7841) | < 0.0001 |
| Negative value (reflux) in Left gonadal vein | | | | |
| SD of LGV | 0.801 (0.711 0.902) | 0.0003 | 0.7140 (0.6322 0.7949) | < 0.0001 |
| MV of LGV | 0.835 (0.754 0.924) | 0.0005 | 0.7031 (0.6220 0.7841) | < 0.0001 |
